# Pulmonary function and thoracic cage morphology during corrective cast treatment of early onset scoliosis

**DOI:** 10.1186/1748-7161-10-S1-O72

**Published:** 2015-01-19

**Authors:** Taichi Tsuji, Noriaki Kawakami, Tetsuya Ohara, Yoshitaka Suzuki, Toshiki Saito, Ayato Nohara, Ryoji Tauchi, Kyotaro Ota

**Affiliations:** 1 Department of Orthopaedic & Spine Surgery, Meijo Hospital, Japan

## Introduction

Historically, corrective casts have been used for the treatment of scoliosis. Over the years, this approach has evolved through countless modifications and improvements. However, while corrective casts are currently used for the treatment of Early Onset Scoliosis (EOS), treatment has been reported to have adverse constrictive effects on the thorax. To date, there have been no studies regarding the effects of corrective casts on pulmonary function and thoracic cage morphology. We hypothesized that cast treatment would have a negative marginal effect on pulmonary function and thoracic cage morphology post-treatment. The purpose of this study was to investigate the inference of the corrective cast treatment on pulmonary function and thoracic cage morphology in patients with EOS.

## Methods

We analyzed the SaO2 and x-ray parameters (i.e., thoracic spinal height, SAL, transverse diameter of thorax, sagittal diameter of thorax) in 14 patients (male: female = 7: 7). Measurements of SaO2 were obtained pre- and post-casting during sleep using a pulse oximeter. The average age was 3.6 years old. The average height and weight were 96.7 cm and 13.8 kg, respectively. Eight (8) patients had syndromic scoliosis, 3 congenital, and 3 idiopathic. The cast was applied for each patient through the use of the Risser table with rotational correction postereolaterally by strap and counter rotation applied on the pelvis, with head halter and pelvic traction.

## Results

The average pre-and post-casting Cobb angles were 56.0 degrees and 28.6 degrees, respectively. The SaO2 was 96.4% prior to casting and 96.9% after casting; the SaO2 did not decrease after casting. While the thoracic spinal height, SAL and transverse diameter of the thorax were increased at post-casting, the sagittal diameter did not change.

## Conclusions

This study did not demonstrate a negative effect of treatment on pulmonary function or thoracic cage morphology. By contrast, a positive effect on thoracic cage morphology was observed. This may have been due to the correction of scoliosis via the cast. Due to the limited number of patients, further research must be conducted with more patient data.

